# The role of Perilipin 5 in pathological myocardial remodeling

**DOI:** 10.3389/fphar.2025.1526494

**Published:** 2025-03-17

**Authors:** Danzeng Dunzhu, Gao Han, Qin Shanshan, Shangshi Li, Jiali Yang, Jian He, Siyu Gou, Gang Dong, Chunrong Jiang, Jun Hou

**Affiliations:** ^1^ School of Medicine, Tibet University, Lhasa, China; ^2^ School of Stomatology, Qilu Medical University, Zibo, China; ^3^ The Department of High Mountain Sickness, The General Hospital of Xizang Military Area Command, Xizang, China; ^4^ School of Life Science and Engineering, Southwest Jiaotong University, Chengdu, China; ^5^ The Third People’s Hospital of Chengdu, Chengdu, China

**Keywords:** myocardial remodel, Perilipin 5, energy metabolism, fatty acids, metabolic disorder

## Abstract

Pathological cardiac remodeling (REM), caused by various pathological factors and characterized by changes in cardiac structure and geometry, is strongly associated with heart failure (HF). It damages cardiac tissue, alters energy metabolism, increases oxidative stress, and cause matrix metalloproteinase activation, cardiomyocyte hypertrophy, and interstitial fibrosis, leading to HF. REM determines the outcome of cardiovascular disease. Current treatments have limitations. REM is associated with cardiac energetic remodeling, and modulation of metabolic substrates may slow down the disease. Perilipin 5 (Plin5), positioned as a structural protein located on the surface of lipid droplets (LDs), is abundant in tissues and cells that rely on mitochondrial β-oxidation for energy production. It is the most recently identified member of the perilipin protein (PAT) family, with a notable enrichment in the cardiac muscle. Emerging evidence highlights the critical role of intracellular LD in the regulation of energy metabolism, with metabolic disruptions of LD being directly correlated with the incidence of metabolic disease. As a key barrier to LD, Plin5 is instrumental in controlling the catabolism of LD and regulating the metabolism and transport of fatty acids (FAs). As a protectant against excessive β-oxidation of free fatty acids (FFAs), Plin5 acts to isolate and neutralize overly oxidized fatty acids, thereby shielding the heart from myocardial remodeling instigated by a variety of etiological factors. This protective mechanism helps to ameliorate the progression of persistent and detrimental myocardial remodeling, which can otherwise lead to the development of severe heart failure. This systematic review attempts to delineate the metabolic disorders associated with pathological cardiac remodeling, focusing on the properties and regulatory mechanisms of Plin5. By synthesising current literature, it investigates the pivotal role of Plin5 in modulating the distinctive attributes, initiating factors, and molecular signaling networks underpinning pathological cardiac remodeling.

## 1 Introduction

**FIGURE 1:**
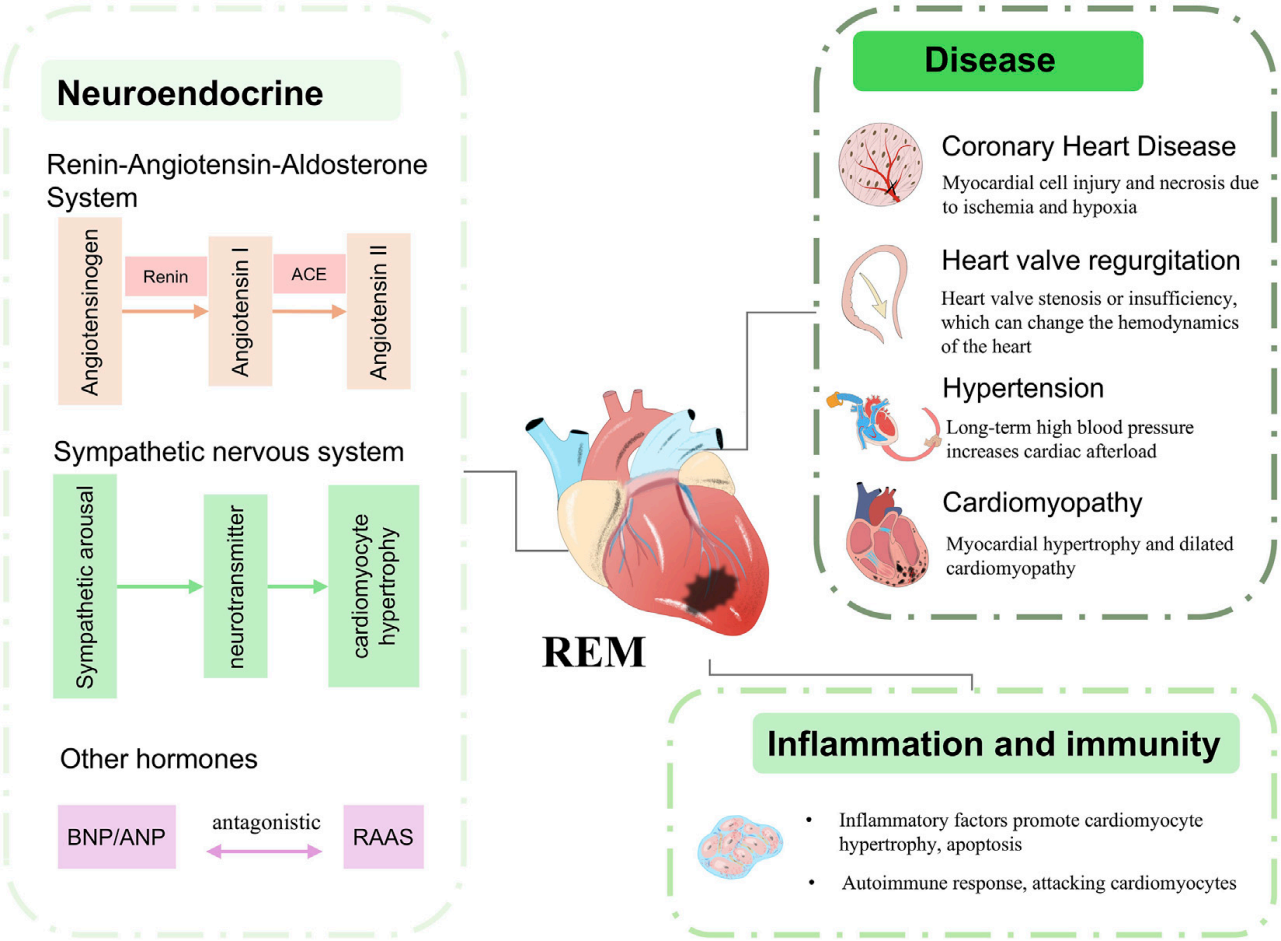
Etiological factors inducing pathological myocardial remodeling.

REM arises from a variety of conditions, including long-term cardiovascular disease such as hypertension, coronary artery disease, dilated or hypertrophic cardiomyopathy, or other underlying conditions such as diabetes mellitus, hyperthyroidism/hypothyroidism, infections, drug toxicity, or genetic influences (Figure 1) changes (Curley et al., 2018; Diez and Ertl, 2008). The above factors contribute to sustained cardiac injury and lead to abnormal cardiomyocyte metabolism, cardiomyocyte apoptosis, TGF-β signalling activation and persistent inflammation, which initiates long-term REM (Gibb and Hill, 2018). In particular, mabnormal cardiomyocyte metabolism affects normal cardiomyocyte function and protein deposition and is one of the most important factors contributing to REM (Cohn et al., 2000). The initial phase of REM represents an “adaptive” response of the heart to injury, dictating the subsequent changes in cardiac geometry and function (Opie et al., 2006). Current medications used for REM therapy aim to reduce cardiac workload or improve myocardial metabolic abnormalities and include beta-blockers, renin-angiotensin-aldosterone system inhibitors, statins, SGLT2 inhibitors and neprilysin inhibitors. Surgical interventions aimed at preventing and reversing remodeling, such as left ventricular assist devices, angioplasty, and biventricular pacing, are often invasive and can cause unnecessary additional harm to patients with cardiovascular diseases (Cokkinos and Pantos, 2011). Emerging therapies involve the infusion of progenitor cells to compensate for the cellular loss caused by remodeling; however, limited clinical trials and meta-analysis results indicate that the therapeutic efficacy of these interventions is currently constrained by several limitations (Glynn et al., 2019).

Traditionally LDs have been viewed as auxiliary organelles within cells that areprimarily tasked with the storage of neutral lipids (Paul et al., 2008). However, recent research is shedding new light on their functions, revealing that LDs are integral to the regulation of vital metabolic pathways. These organelles serve as reservoirs for fatty acids (FAs), which are essential as primary energy substrates in metabolic processes (Zheng et al., 2017). The heart, which is endowed with a robust capacity for β-oxidation, derives approximately 50%–70% of its energy requirements from the oxidation of FAs contained within LDs under physiological conditions. The remainder of the energy is supplied through the oxidation such as glucose, lactate, and pyruvate. Nevertheless, excess FA oxidation (FAO) is associated with increased oxygen consumption. Typically, myocardial cells transform surplus FAs into triglycerides (TGs) for storage within LDs. Nevertheless, an excess of FFAs can lead to lipotoxicity, a condition detrimental to cellular health (Walther and Farese, 2012).Current evidence suggests that disturbances in energy metabolism serve as the main pathological basis for REM, with the key indicator of such disruptions being the malfunction of FA metabolism (Barth and Tomaselli, 2009; Ezeani, 2020). LDs are equipped with a large number of regulatory proteins associated with lipid droplet function, the most prominent of which is the PAT. Members of this family are located on the surface of LDs and are collectively referred to as perilipins or peridroplet proteins. Previous studies have shown that Plin5 plays a direct role in the regulation of LD metabolism and is highly enriched in cardiac tissue. By inhibiting the lipolysis of LDs, Plin5 can reduce lipid oxidation. Recent research has investigated the impact of Plin5 on myocardial remodeling by modulating its expression levels (Kimmel et al., 2010). Considering that myocardial remodeling can be triggered by a variety of etiological factors and that there is a current gap in systematic reviews concerning Plin5, the objective of this research is to assess whether modulating Plin5 levels might diminish the probability of cardiac remodeling.

In this review, we first take a retrospective look at the unique characteristics of energy metabolism changes in REM, which include both the remodeling of energy metabolism and the reconfiguration of mitochondrial function. We then provide a synthesis of the current understanding of Plin5’s role and the underlying mechanisms in fatty acid metabolism. Building upon this framework, we delve into the manner in which Plin5 may regulate REM and conduct an exhaustive examination of the mechanisms linking the energy metabolism of myocardial remodeling to Plin5. Finally, we highlight the critical areas that merit further investigation in upcoming research efforts.

## 2 The energy metabolism of REM

REM is considered a critical step in the progression of HF, characterised by significant pathological changes such as myocardial hypertrophy, fibrosis, and inflammatory responses (Feng et al., 2018). Despite the numerous potential causes of cardiac injury, each one tends to activate a spectrum of myocardial remodeling to some extent. This remodeling process is governed by the renin-angiotensin-aldosterone system (RAAS), catecholamines, cortisol, and genetic influences, which collectively result in a diminished flexibility of energy metabolism (La et al., 2009). The hallmark of this metabolic shift is the alteration in the metabolism of fatty acids and glucose substrates. Such metabolic remodeling, in turn, promotes widespread structural changes within the heart (Chandler et al., 2004).

The heart preserves tissue equilibrium and supports biosynthetic processes by finely tuning its energy metabolic pathways, thereby controlling cell phenotype and managing redox reactions (Gibb and Hill, 2018). Metabolic intermediates and end-products serve as signaling molecules that modulate enzyme activity and influence gene expression patterns. The substantial energy requirements of cardiac are largely satisfied through oxidative phosphorylation, while a smaller fraction is obtained via glycolytic phosphorylation. In normal physiological conditions, mitochondria rely on FA as key metabolic substrates to energize the myocardium, with the heart’s preference for metabolic substrates being predominantly governed by hormonal and plasma substrate concentrations, such as those of insulin and catecholamines. During myocardial remodeling, the heart increases its reliance on glucose as a fuel. Metabolic changes during development, exercise, and pregnancy, as well as pathological states such as myocardial infarction, ischemia/reperfusion, pulmonary hypertension, and diabetes, can induce energy metabolic disorders that are triggers for myocardial remodeling (Gibb and Hill, 2018). In the early stages of cardiac remodeling, changes in energy metabolism can protect the heart from irreversible injury. However, as the disease progresses, energy deficiency leads to changes in metabolic substrate preference related to mitochondrial abnormalities and difficulties in ATP transport, collectively causing energy metabolic impairment in myocardial remodeling (Azevedo et al., 2013). Evidence suggests that in myocardial remodeling, metabolic remodeling and mitochondrial injury precede structural remodeling of the heart and are associated with cardiac contractile dysfunction (Sharma et al., 2007). The energy remodeling in REM is primarily due to a decrease in high-energy phosphate content resulting from altered substrate utilization and energy metabolism in the heart. Metabolic remodeling and mitochondrial dysfunction are key indicators of the progression of REM to cardiac hypertrophy and failure (Azevedo et al., 2013).

### 2.1 Variations in energy metabolism substrates

The substrates required for cardiac metabolism primarily include endogenous sources (Crass et al., 1975) (triglycerides, glycogen) and exogenous carbon substrates (Gousios et al., 1965) [FAs (Buse et al., 1973), glucose, pyruvate, branched-chain amino acids]. The high adaptability of cardiac metabolism to substrate supply and demand is crucial for maintaining heart function during both development and pathological states. Under physiological conditions, cardiac mitochondria mainly produce ATP through the oxidation of fatty acid acyl-CoA (CoA) and pyruvate, which are the primary metabolic products of fatty acids and carbohydrates, respectively. Current research indicates that changes in substrate preference are observed in various cardiovascular-related diseases (Sansbury et al., 2014). Long-term exposure to increased fat intake, abnormal liver fat synthesis, and lipolysis can lead to elevated levels of circulating FFA and triglycerides (TG). Initially, the body can store excess FA as TG, but excess lipid accumulation is eventually diverted to non-oxidative pathways, leading to the buildup of toxic lipids. This alters cellular signaling, promotes mitochondrial dysfunction, activates oxidative stress pathways, and induces massive cell apoptosis. Increased glucose oxidation can compensate for the loss of fatty acid oxidation capacity due to pathological myocardial remodeling. However, “glucose substrate dependency” often signifies reduced oxidative metabolic capacity and high glycolytic rates. Numerous studies (Chokshi et al., 2012) have shown that after myocardial remodeling occurs, due to mitochondrial injury and other factors, the rates of glycolysis and glucose oxidation are lower (Ae et al., 2010). At the same time, due to the limited supply of ketone bodies and amino acid substrates, their contribution to cardiac oxidative metabolism is minimal.

The changes in myocardial metabolism are influenced by a variety of factors, including oxygen supply, coronary perfusion substrate delivery, hormone concentrations, positive inotropy status, and tissue nutritional state. Relative to a healthy or structurally normal heart, the ratio of phosphocreatine to ATP (PCr-ATP) in a remodeled heart is altered. Current evidence suggests that changes in cardiac energy substrate metabolism can modulate multi-pathway metabolites, which act as signaling molecules, thereby inducing changes in myocardial cell performance and cardiac function (Stefan, 2007).

#### 2.1.1 Fatty acid metabolism

Current evidence strongly supports the close relationship between the occurrence of various metabolic diseases and dyslipidemia (Azevedo et al., 2013). Conditions such as obesity, diabetes, and dyslipidemia are associated with a significantly increased risk of cardiovascular disease. Post-cardiac injury, there is an upsurge in catecholamines and cortisol levels, which promote lipolysis and result in enhanced myocardial fatty acid uptake. Nevertheless, because fatty acids necessitate 10%–12% more oxygen than glucose to generate the same quantity of ATP, this increased demand leads to further oxygen consumption and intensifies cardiac injury.

Relevant studies have demonstrated that dysregulation of lipid homeostasis exacerbates the progression of adverse myocardial remodeling (Schaffer, 2003). The provision of FA to the heart is derived from plasma-free fatty acids and triglyceride-rich lipoproteins, which are hydrolyzed under the action of lipoprotein lipase (LPL) and endogenous TG (Bergman et al., 2009). As intermediate products in lipid metabolism, FAs account for approximately 60%–90% of myocardial ATP production under physiological conditions, with the resultant products being FA-acyl-coA. This is further processed through the FA oxidation spiral cycle to generate acetyl-CoA, NADH, H_2_O, and FADH_2_ (van der Vusse et al., 1992).

FAs are primarily classified into triglycerides, phospholipids, and cholesterol esters, with triglycerides existing in a stable form within low-density lipoproteins (LDL). Under physiological conditions, the heart exhibits limited storage of LDL. However, in the context of comorbidities such as diabetes, obesity, and cardiovascular-related disease, the heart has been found to contain an abundance of larger LDL particles (Goldberg et al., 2018). When FAs are not present in an esterified form in circulating plasma, they are referred to as non-esterified fatty acids (NEFAs) or FFA.

**FIGURE 2:**
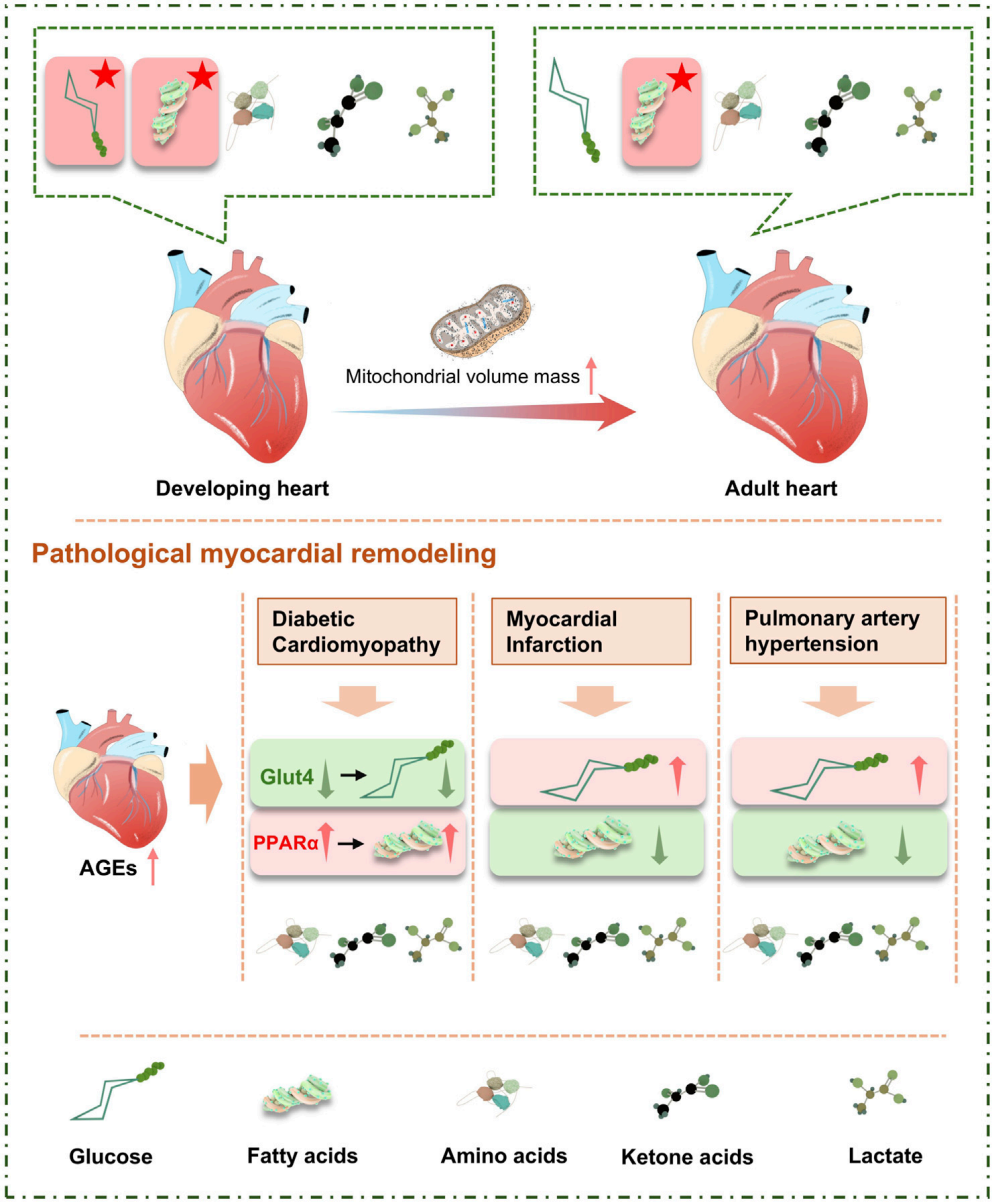
Metabolic characteristics and substrate changes in the heart.

FFA are transient, constituting only 5%–10% of total serum fatty acids under normal conditions. During fasting, stress, or exercise, when energy demands increase, FFA are activated by acyl CoA synthetase (ACS) to form acyl-CoA. Among these, medium-chain acyl-CoA esters are passively absorbed by mitochondria-containing cells from the circulating plasma, while 75% of long-chain acyl-CoA esters are mediated by carnitine palmitoyltransferase 1 (CPT-1) and transported into the mitochondria (Voros et al., 2018). Here, they are oxidized and enter the citric acid cycle to release energy in the form of ATP. Besides oxidation in the mitochondria, acyl-CoAs can also be converted into ceramides, diacylglycerols (DAG), and triglycerides (TAG) (Figure 2).

The formation of DAG can also occur through the hydrolysis of phospholipids, leading to the activation of protein kinase C (PKC). Therefore, FFA are utilized as an energy source for tissues such as the liver, muscle, and heart, while excess free fatty acids are re-esterified into triglycerides for storage. However, a small fraction of hydrolyzed FFA may escape and contribute to the general plasma FFA pool. Unoxidized FAs are stored in triglycerides and ceramides, with DAG and ceramides increasing in reconstructed hearts. The transfer of these toxic lipid intermediates to the triglyceride reservoir can prevent lipotoxicity. Triglycerides are hydrolyzed by adipose tissue triglyceride lipase (ATGL), and the resulting fatty acids can enter the fatty acid oxidation pathway or serve as ligands for nuclear receptor activation.Relevant findings indicate that excessive lipid accumulation within the myocardium leads to the inhibition of Akt1 (Ritterhoff and Tian, 2017).

FAs also play crucial roles in receptor signaling, gene expression, and the regulation of systemic fuel energy homeostasis under various physiological conditions. Studies have confirmed that elevated levels of FFA lead to the onset and progression of diseases such as metabolic syndrome, atherosclerosis, acute coronary syndrome, and heart failure. The stimulation by FFA results in an increase in the production of reactive oxygen species (ROS) and reactive nitrogen species (RNS), triggering oxidative stress mechanisms—characterized by a long-term imbalance between the production of reactive species and antioxidant defenses, leading to tissue injury. These reactive molecules can directly oxidise and damage DNA, proteins, and lipids, and they can also act as functional molecular signals to activate a variety of stress-responsive signalling pathways within cells.

Two potential sources of fatty FA reach the heart: circulating non-esterified FAs (NEFAs) bound to plasma albumin and circulating esterified FAs in the form of triglycerides (TGs) and cholesterol esters (CEs) transported by large lipoproteins containing TGs, including residual chylomicrons and very low-density lipoproteins (VLDL). Under normal conditions, the primary energy source for myocardial cell contraction metabolism is free fatty acids (FFAs), with the heart’s uptake of FFA primarily determined by the concentration of non-esterified fatty acids. Non-esterified long-chain FAs are the main metabolic source for the myocardium, accounting for nearly two-thirds of the ATP produced, while the remaining one-third of the myocardial oxygen demand is met by glucose metabolism. Plasma long-chain fatty acids are either esterified to glycerol or remain non-esterified (or FFA), with the majority bound to albumin. Myocardial cells utilize β-oxidation of free fatty acids to provide energy, reduce glucose utilization, and increase glycogen stores, which is important for myocardial cells. This energy supply pattern not only provides energy but also consumes potentially “lipotoxic” free fatty acids, thereby reducing the risk of heart disease.

Excess FFA has a destructive effect on cardiac tissue, leading to a range of myocardial dysfunctions, including accumulation of toxic intermediates of fatty acid metabolism, inhibition of glucose utilization, generation of reactive oxygen species, oxygen depletion and increased insulin resistance. The activation of the pro-inflammatory transcription factor Nuclear Factor Kappa B triggers oxidative stress mechanisms, upregulates inflammatory mediators, and damages endothelial cell function. The ultimate result is the acceleration of heart failure progression (Sharma et al., 2004).

The downregulation of FAO expression may be partially due to a decrease in the transcriptional activity regulated by the PPARα/RXRα/PGC-1α heterotrimer (Huss and Kelly, 2004). PPAR is a key regulator of FAO-related genes, with the lipid metabolism transcription factor PPARα highly enriched in the heart. Activation of PPARα by upregulating FA metabolism-related genes promotes fatty acid uptake, utilization, and catabolic metabolism. PPARα knockout (KO) mice exhibit severely impaired FAO and high glucose oxidation; conversely, PPARα transgenic mice show increased fatty acid uptake and oxidation capacity, with reduced glucose oxidation, ultimately leading to cardiac hypertrophy and heart function impairment (Ritterhoff and Tian, 2017). Overexpression of PPARα to increase fatty acid uptake and oxidation results in cardiomyopathy, while specific enhancement of mitochondrial FAO can improve pathological hypertrophy and heart dysfunction caused by myocardial remodeling. Similar to PPARα, PPARβ/γ TGs exhibit upregulation of genes related to fatty acid and glucose metabolism, improving myocardial energy metabolism conditions under myocardial remodeling (Liu et al., 2011).

#### 2.1.2 Glucose metabolism

Glucose metabolism primarily occurs via the pentose phosphate pathway, and the substrates required for endogenous cardiac glucose metabolism are derived from the glycogen stored within the heart. It is characterised by its ability to be rapidly metabolized to rapidly turnover to facilitate energy supply. Myocardial cells absorb glucose mainly through GLUT-1 and GLUT-4 receptors, which are both subtypes of GLUT expressed in the heart. GLUT-1 is primarily responsible for mediating insulin-independent glucose transport, while GLUT-4 mediates insulin-sensitive glucose transport (Wc et al., 2005). The terminal products of oxidative glycolysis are phosphopentose ketone acid and the reduced coenzyme NADP+ (NADH), whereas the end products of non-oxidative glycolysis are phosphopyruvate. These intermediates are transported into the mitochondrial matrix for energy metabolism. Pyruvate is oxidized to form acetyl-CoA, a process regulated by the pyruvate dehydrogenase (PDH) complex. During the initial phase of remodeling, Akt-1 and AMPK mediate the translocation of GLUT to the cell surface, leading to increased glucose uptake (Shao and Tian, 2015). Current evidence suggests that glucose utilization and enhanced glycolysis contribute to disease progression and chronic cardiac remodeling. When cardiac metabolism is impaired, the rate of glucose uptake exceeds the heart’s capacity for glucose oxidation, resulting in the accumulation of a key intermediate, glucose-6-phosphate (G6P), which can enter the glycolytic, hexosamine biosynthesis pathway (HBP), or the pentose phosphate pathway (PPP). This accumulation also mediates the activation of the mTOR pathway, whose activation impairs cardiac function and induces endoplasmic reticulum stress responses (Sen et al., 2013).

The enhanced glucose utilization is not attributed to an upsurge in oxidative glycolysis, but is instead a consequence of the downregulation of FAO (Danilo et al., 2007), which acts as a competitive substrate. This is compounded by the diminished activity of pyruvate dehydrogenase, the pivotal rate-limiting enzyme in glucose oxidation, along with a decline in the overall mitochondrial oxidative capacity, leading to a relative decrease in glucose availability within the cardiac mitochondria (Wg et al., 2014). Liao et al. (2002) demonstrated that the cardiac-specific overexpression of GLUT-1 in transgenic mice significantly enhanced basal myocardial glucose uptake compared to wild-type controls. This overexpression markedly delayed the progression towards heart failure and extended the overall survival of the mice. Nonetheless, it is crucial to recognize that the glucotoxicity induced by high glucose metabolism, in conjunction with elevated levels of free fatty acids (FFA), can further aggravate cardiac toxicity (Peter et al., 2002).

#### 2.1.3 Ketone body metabolism

Ketone bodies are produced by the liver and utilized for energy metabolism in the heart and brain during conditions of nutrient deprivation (Aubert et al., 2016). Within the mitochondria, ketone bodies are rapidly converted to acetyl-CoA through a series of reactions catalyzed by β-hydroxybutyrate dehydrogenase (BDH1), succinyl-CoA:3-oxoacid CoA transferase (SCOT), and mitochondrial acetyl-CoA acetyltransferase 1 (ACAT1). There is evidence that circulating ketone body levels are elevated in patients with advanced heart failure.

Ketone bodies compete with other metabolic substrates, particularly fatty acids, and the shift towards ketone body oxidation occurs in the context of reduced fatty acid oxidation as the primary substrate in the adult heart. This shift is predicted to be driven by changes in the transcription of genes related to fatty acid utilization, mediated by peroxisome proliferator-activated receptor alpha (PPARα), as part of the energy metabolic remodeling. Studies in rodent models have confirmed that the hearts of animals with myocardial hypertrophy and advanced heart failure use ketone bodies as a compensatory fuel for ATP production (Lahey et al., 2014; Sack et al., 1996). By targeted disruption of the key enzyme SCOT in the ketone body oxidation pathway, it was found that rodents with pressure overload developed a heart failure phenotype at an earlier stage (Schugar et al., 2014).

As myocardial remodeling progresses and heart failure approaches its terminal stage, the oxidation of pyruvate is reduced and most is converted to lactate, leading to a decrease in intracellular pH. This acidosis further contributes to impaired myocyte contraction.

#### 2.1.4 Branched-chain amino acid metabolism

Branched-chain amino acids (BCAAs) consist of leucine, isoleucine, and valine, which share a common structural feature in their side chains and possess a shared catabolic pathway (Huang et al., 2011). BCAAs are transported into cells via specific amino acid transporters and are subsequently catabolized within the mitochondria, ultimately being oxidized in the tricarboxylic acid (TCA) cycle. Under physiological conditions, the energy contribution from BCAAs is minimal, and their catabolism primarily occurs in non-hepatic tissues such as the brain, heart, and kidneys (McGarrah and White, 2023). BCAAs are converted to branched-chain α-keto acids (BCKAs) by the mitochondrial branched-chain aminotransferase (BCATm) (Harris et al., 2004). BCKAs are then oxidized by the branched-chain α-keto acid dehydrogenase (BCKD) complex, ultimately leading to the degradation into acetyl-CoA or succinyl-CoA. The normal catabolism of BCAAs is essential for maintaining cardiac physiology and cellular viability. Previous evidence has indicated that variations in plasma BCAA levels are associated with cardiovascular and metabolic diseases (Sun et al., 2016).

BCAAs act concurrently as potent signaling molecules and as effective activators that regulate mTOR activity through signaling pathways, leading to the inhibition of autophagy upon mTOR activation triggered by BCAAs (Nicklin et al., 2009). The increase in BCAAs can induce cytotoxicity. Simultaneously, BCAAs are involved in the regulation of several cellular processes, including insulin signaling, protein translation, and autophagy, and they also impact the metabolism of glucose and FAs (Melnik, 2012). During metabolic remodeling in pathological hearts, a compromised capacity for BCAA catabolism results in elevated local concentrations of BCKA metabolites, leading to an increase in cardiac mTOR activity. Chronic activation of mTOR, induced by BCAAs, suppresses the cardioprotective autophagic capacity, leading to mitochondrial dysfunction. This dysfunction is manifested by the loss of mitochondrial membrane potential (ΔΨm), ROS accumulation, and the opening of the mitochondrial permeability transition pore (mPTP), which induces myocardial hypertrophy and contributes to cardiac structural remodeling (Huang et al., 2011).

Significant metabolic dysregulation of branched-chain amino acids (BCAA) has been observed in the hearts of mice subjected to pressure overload and MI. Genetic defects in BCAA catabolism can lead to Maple Syrup Urine Disease (MSUD). The BCAA catabolic pathway is regulated by Krüppel-like factor 15 (KLF15), which is considered a primary transcriptional factor for BCAA. KLF15 plays a functional role in regulating myocardial hypertrophy, heart failure, and myocardial fibrosis (Sun et al., 2016). Additionally, KLF15 acts as a critical regulator of other metabolic substrates, such as glucose and FAs (Haldar et al., 2012). The expression level of KLF15 is significantly reduced in the myocardium of pressure overload cardiomyopathy. In summary, KLF15 is an important regulatory factor in the metabolic remodeling of the heart (Prosdocimo et al., 2014).

The substrate shifts in cardiac metabolism reflect a reversion from adult to fetal metabolic phenotype, ensuring that the heart is protected from irreversible and fatal structural and functional deterioration when the “organ engine” faces insufficient energy supply (Taegtmeyer et al., 2010). Pathological hearts have exhibit a reduced capacity for fatty acid oxidation and compensate by using the glycolysis/oxidation pathway for the pentose phosphate pathway. The adverse consequence of this metabolic shift is the impairment of stress response and subsequent impact on myocardial mitochondrial function (Gupte et al., 2007).

Post-myocardial infarction, there is a reduction in the supply of oxygen and metabolic substrates to the heart, which diminishes FAO (Jiang et al., 2021). This decrement activates anaerobic glycolysis, leading to the production of lactate—a less efficient energy source for the heart. Chronic lactate accumulation inhibits glycolytic enzymes and causes a decrease in pH, thereby triggering apoptosis of myocardial cells. In the context of diabetic cardiomyopathy, myocardial remodeling is marked by metabolic changes involving reduced glucose oxidation and enhanced fatty acid uptake and oxidation. The increase in FAO directly results in diminished oxygen efficiency. Both diabetic and hyperobese patients exhibit significant lipid accumulation within the myocardium, which correlates positively with cardiac dysfunction and can evolve into lipotoxic cardiomyopathy. The imbalance between lipid supply and oxidation rates leads to a downregulation of FAO, ultimately compromising cardiac contractility (Kolwicz et al., 2013). Research has demonstrated that interventions aimed at reducing fatty acid uptake or enhancing cardiac lipid storage capacity can improve the condition of lipotoxic cardiomyopathy (Liu et al., 2009).

### 2.2 Abnormal mitochondrial function in the myocardium

**FIGURE 3:**
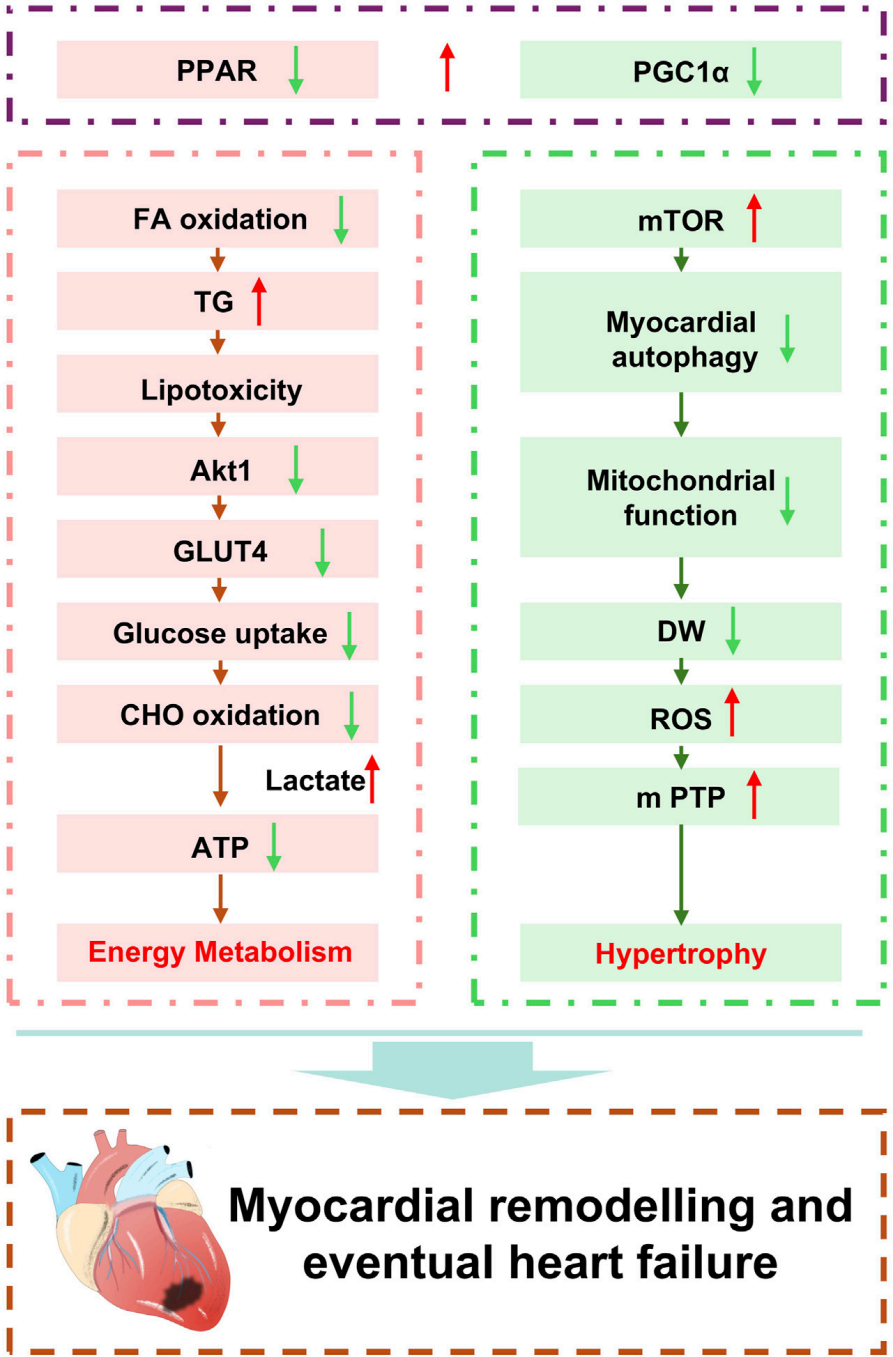
Mechanistic insights into pathological myocardial remodeling.

Myocardial mitochondria serve as the energy hub of cardiomyocytes, responsible for the synthesis of the ATP required by the cell (Chang et al., 2023). The myocardium contains a high density of mitochondria to meet the unique energy demands of driving myocardial contraction and maintaining ionic homeostasis. The structure, function, and metabolic level of mitochondria are all critically important for maintaining normal cardiac metabolism. During myocardial remodeling, cardiomyocytes are subjected to various stimuli and injuries, leading to mitochondrial dysfunction. In the process of pathological myocardial remodeling, which is accompanied by diastolic and systolic dysfunction, systolic dysfunction often coincides with mitochondrial injury, suggesting a significant impact of mitochondria on contractile function (Figure 3).

#### 2.2.1 Impaired energy metabolism

Cardiomyocytes depend on contractile proteins to sustain their function, with mitochondrial oxidative phosphorylation being crucial for the maintenance of cardiac systolic and diastolic functions. Mitochondria produce ATP by oxidizing glucose, FAs, and amino acids, releasing energy for oxidative phosphorylation, with the terminal common pathway being the tricarboxylic acid cycle (Fernie et al., 2004). However, the uptake of fatty acids may exceed the mitochondrial capacity for fatty acid oxidation, leading to an increase in cardiac lipid storage rather than oxidation metabolism. Excessive delivery of fatty acids to the myocardial tissue results in enhanced FA uptake and oxidation, with excessive mitochondrial beta-oxidation causing an increase in production of ROS.

This initiates a cascade of reactions, with the initial surge of ROS inducing mitochondrial uncoupling. This may lead to an increase in mitochondrial oxygen consumption (V̇O2), thereby further promoting fatty acid oxidation. Since the increase in V̇O2 is a result of mitochondrial uncoupling, ATP synthesis does not increase proportionally. Simultaneously, ROS may also induce oxidative injury to mitochondrial proteins involved in energy metabolism and oxidative phosphorylation, which can further affect ATP synthesis.

Ultimately, the reduction in ATP synthesis may fail to meet the energy demands of the myocardium, leading to contractile dysfunction. The progression of excessive lipid accumulation can eventually result in dysregulation of myocardial energy metabolism. *In vitro* experiments with isolated perfused hearts have demonstrated that high concentrations of FFA can increase oxygen consumption and decrease myocardial contractility, confirming the process. These findings indicate that dysregulation of FFA metabolism can impact the production and utilization of ATP in cardiomyocytes. The rise in the ratio of low fatty acid metabolism to glycolysis results in myocardial lipotoxicity, acidosis, and reduced ATP generation (Azevedo et al., 2013). The lack of a precise relationship between endoplasmic reticulum (ER) stress and the structure and function of the mitochondrial system may be associated with lipid-induced dysfunction of the heart.

Dysregulation of energy metabolism directly impacts cardiac function. Manipulating fuel supply and substrate consumption appears to compensate for the energy metabolic disturbances under pathological conditions (Revenco and Morgan, 2009). Current research supports therapeutic strategies for pathological myocardial remodeling by increasing the utilization of fatty acids or ketones, reducing the carbon derived from glucose sources for anabolic metabolic reactions, to prevent or delay pathological myocardial remodeling (Berthiaume et al., 2010).

#### 2.2.2 Increased oxidative stress

In pathological conditions, mitochondria experience C overload and the accumulation of ROS, leading to alterations change in tissue homeostasis and the loss of cardiomyocytes. The initial phase of cardiac injury is characterized by a robust inflammatory response, in which NO signaling plays a role. Due to the reduced expression of endothelial nitric oxide synthase (eNOS), there is an increased production of superoxide anions. Excessive NO interacts with superoxides to generate potent reactive oxygen species (peroxynitrites). Given the high number of mitochondria within the myocardium, significant production of ROS occurs, leading to oxidative stress and changes in cellular membrane permeability. This further results in mitochondrial DNA (mtDNA) injury and a decline in mitochondrial function, affecting mitosis and mitochondrial fission/fusion (Zhou et al., 2019). Repeated accumulation of ROS, through the activation of matrix metalloproteinases (MMPs), induces myocardial hypertrophy, apoptosis, and matrix fibrosis, which are cellular precursors leading to pathological myocardial remodeling and heart failure.

Metabolic imbalance in cardiomyocytes leads to the accumulation of ROS within mitochondria, resulting in an increase in mitochondrial membrane potential and stimulation of the opening of mitochondrial permeability transition pores (mPTPs). Concurrently, overactivation of oxidative phosphorylation and the tricarboxylic acid cycle leads to abnormal elevations in ATP and ROS levels, triggering mitochondrial dysfunction and oxidative stress injury (Kolwicz et al., 2013). Chronic pathological stimulation activates the AMPK pathway, where AMPK activation can increase mitochondrial autophagy and inhibit apoptosis pathways in cardiomyocytes (Zhang et al., 2017). Oxidative stress has a direct impact on cellular structure and function, potentially acting as a global signaling molecule that directly activates myocardial remodeling (Tsutsui et al., 2009). Prolonged dysregulation leads to the clearance of numerous damaged mitochondria by mitosis, and dysregulation of the mitotic pathway also contributes to further ROS accumulation. Multiple mechanisms interact with each other, exacerbating mitochondrial oxidative stress responses, and ultimately leading to cardiomyocyte death (Zhu et al., 2018).

#### 2.2.3 Mitochondrial DNA mutation

The pivotal mediator in cardiac mitochondrial dysfunction, peroxisome proliferator-activated receptor gamma coactivator-1α (PGC-1α), NRF1/2 (nuclear respiratory factors), and mitochondrial transcription factor A (Tfam), are considered to be responsible for the impairment of cardiac mitochondrial function. PGC1α/β is primarily expressed in high-oxidation tissues and acts as a coordinator in acute metabolic disturbances by regulating the expression of nuclear genes encoding mitochondrial fatty acid oxidation enzymes and controlling mitochondrial biogenesis. The absence of PGC1α and β leads to a decrease in the expression of genes encoding mitochondrial oxidative enzymes. Mitochondrial function is controlled by mitochondrial mtDNA and its transcriptional regulatory factors, which together contribute to the possibility of mtDNA damage and impaired mitochondrial gene transcription and/or replication in myocardial remodeling. Depletions and mutations in mtDNA are closely associated with mitochondrial dysfunction, and studies have found that in children with congenital heart disease, reduced mtDNA replication and deletions accelerate the transition from myocardial hypertrophy to heart failure (Karamanlidis et al., 2011). Similarly, decreased mtDNA copy numbers after myocardial infarction also predict heart failure. Clinical research suggests that under conditions of myocardial remodeling, mtDNA mutations accelerate the progression of heart failure, and changes in mitochondrial mtDNA are sufficient to induce myocardial remodeling.

The interaction between the mitochondrial biogenesis proteins PGC1α and Nrf1/2 plays a crucial role in regulating mitochondrial biogenesis and mitochondrial homeostasis (Wu et al., 2021). Their expression is significantly suppressed in the hearts under pressure overload, and evidence indicates that they can affect the development and maturation of cardiomyocytes and the synthesis of mtDNA, thereby controlling the life processes of cardiomyocytes. Pathological myocardial remodeling involves dysregulation of mitochondrial dynamics, autophagy, and biogenesis, which impact the metabolism and survival of cardiomyocytes (Chang et al., 2023).

## 3 Perilipin 5: regulation of fatty acid metabolism function and mechanisms

### 3.1 Structural characteristics and expression profiling of Perilipin 5

Since the 1980s, members of the PAT (Perilipin, ADFP, and TIP47) family have been sequentially identified, named after the highly phosphorylated lipid droplet protein associated with PKA, adipose differentiation-related protein (ADFP), and tail-interacting protein (TIP47) (Paul et al., 2008). Subsequently discovered members include S3-12 and OXPAT. OXPAT is also known as myocardial lipid droplet protein (MLDP), lipid droplet storage protein 5 (LSDP5), and Perilipin 5, which is the focus of this study. For precise description, this manuscript refers to all members collectively as Perilipin 1 to 5.

The PAT protein family exhibits structural similarities, with Plin 5 sharing a conserved homologous region with Plin 2 at the C-terminus. The C-terminal regions of Plin 2 and Plin 3 are alpha-helical, comprising an alpha-beta domain and a 4-helix bundle (Sztalryd and Kimmel, 2013). Except Plin 4, the other four members share a highly conserved N-terminal domain known as the PAT (perilipin, ADFP, and TIP47) domain. Plin proteins also possess a conserved tandem repeat sequence of 11-mer units and exhibit considerable similarity in other regions. Plin proteins typically appear to protect LDs from lipase attack, although their efficiency, distribution, and regulatory mechanisms vary.

### 3.2 The role of Perilipin 5 in fatty acid metabolism

The formation of LDs is a pivotal aspect of cell physiology, and an increasing body of research indicates that their function extends beyond mere energy storage, with their multifunctionality gaining broader recognition. LDs maintain lipid homeostasis by regulating lipid synthesis, metabolism, and transport, effectively preventing the accumulation of toxic lipids (Wang et al., 2013). Simultaneously, LDs can play roles in energy metabolism, membrane synthesis, and the provision of lipid-derived signaling molecules. Current research suggests that LDs are initially produced by enzymes in the endoplasmic reticulum, undergoing processes of neutral lipid synthesis, nucleation, cytoplasmic budding, and growth. Rich in triglycerides, LDs provide the largest storage reservoir of esterified fatty acids for energy, which are mobilized to skeletal muscle, myocardium, and other tissues of the body for β-oxidation metabolism to support ATP production (Wang et al., 2013). Proteins involved in LD formation or maintenance are crucial for normal cell physiology, and mutations in specific LD proteins can lead to cellular dysfunction, severe metabolic disturbances, and diseases. Evidence suggests that defects in lipid droplet packaging are closely associated with severe heart failure (Chokshi et al., 2012). Plins are the most abundant class of proteins on the surface of LDs, with Plin 5 widely expressed in high metabolic tissues (Cinato et al., 2024). Under physiological conditions, Plin 5 maintains LD synthesis and degradation by restricting lipase activity. Experimental designs using Plin 5 knockout animal models have shown that the absence of Plin 5 leads to the accumulation of LDs over time. The long-term accumulation of lipids can result in cardiac toxicity, including the build-up of ceramides, long-chain acyl-CoAs, and acylcarnitines. LDs can prevent cardiac lipotoxicity by storing FAs in the non-toxic form of triglycerides, thus avoiding myocardial remodeling and heart failure (Guo et al., 2022).

LDs harbor hydrophobic cores consisting of TAG and cholesterol esters (CE), which are synthesized in the endoplasmic reticulum (ER). The core component of these droplets is FA. Activation of FA leads to the production of diglycerides or cholesterol, which are subsequently transformed into TAG and CE by distinct enzymes within the ER, thus completing the assembly of new lipid droplets (Karanasios et al., 2010).

**FIGURE 4:**
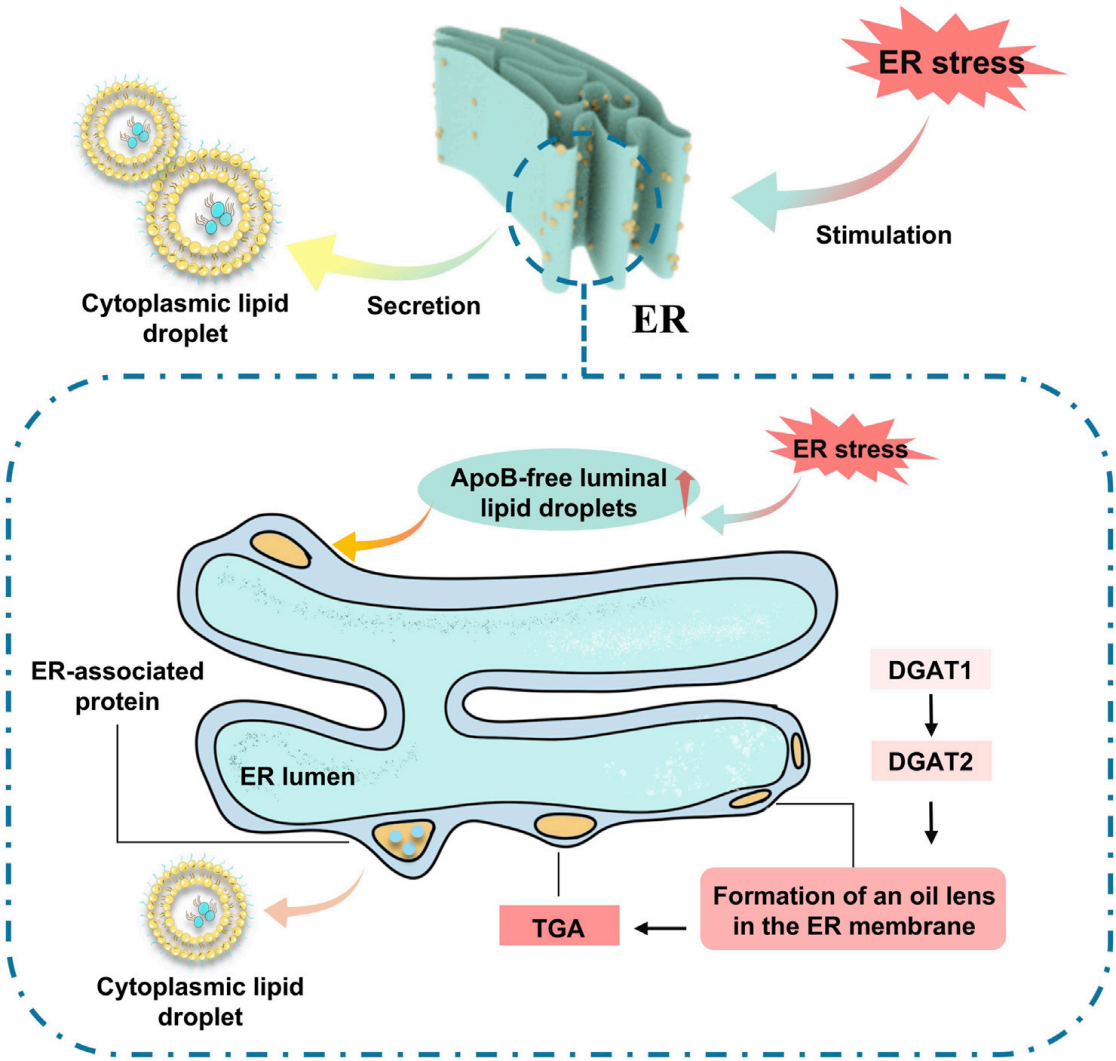
Endoplasmic reticulum-mediated lipid droplet formation.

Working cells adapt to environmental changes by transitioning their metabolic energy source from a glucose-dependent to a mitochondrial FA oxidation-dependent state. This adaptation is particularly pronounced in cardiomyocytes, which have a substantial energy requirement and thus favor FA oxidation as an energy source. During nutritional stress, cells facilitate the transport of FAs by autophagic digestion of membrane-bound organelles (ER) or lipid droplets. Additionally, they can catabolize LDs through hydrolysis by neutral lipases. Although these mechanisms can swiftly satisfy the cytoplasmic demand for FAs, the increase in free FAs, due to some FAs not being immediately taken up by the mitochondria for energy conversion, inevitably results in lipotoxicity, which may subsequently initiate related pathologies (Figure 4).

FA are enzymatically degraded during nutritional stress to preserve cellular energy levels. This necessitates the mitochondrial import of FAs to yield metabolic intermediates that power respiration. β-Oxidation, the primary pathway for FA metabolism, takes place within the mitochondrion. As fatty acids are hydrolyzed to fulfill cellular energy demands, mitochondrion plays a pivotal role in producing intermediates that drive the respiratory cycle. Cells upregulate the intake of mitochondrial FAs and the activity of lipases that mediate β-oxidation, leading to an increase in intracellular FFA. These FFA act as ligands to activate PPARs, which in turn induce the expression of Plin5. Plin5 interacts with FFA metabolism, exerting a regulatory feedback (Lockridge et al., 2008).

In basal conditions, the breakdown of fats is primarily regulated by the translocation of lipases from the cytoplasm to the surface of LDs (Zhang et al., 2022). Studies have indicated that Plin5 restricts the entry of lipases (Brasaemle et al., 1997), such as hormone-sensitive lipase (HSL) and adipose triglyceride lipase (ATGL), into the core of triglycerides within LDs, thus preventing uncontrolled hydrolysis and limiting the release of free fatty acids (FFAs) and glycerol. Alpha-beta hydrolase domain-containing 5 (ABHD5) (Granneman et al., 2010), an activator of ATGL lipase activity, interacts with Plin5, which in turn inhibits lipolysis by binding to both ATGL and ABHD5. Plin5’s oligomerisation ability allows for the close interaction between ATGL and CGI-58.

When cells experience a sudden increase in nutritional demands, FAs are cleaved by enzymes to supply energy. The binding of ATGL and CGI-58 to Plin5 is competitive, with Plin5 binding to ATGL and CGI-8 separately, functioning as a positive regulator of CGI-8. Mitochondrial fatty acid oxidation is a crucial energy source for the heart. Animals with Plin5 overexpression show a marked increase in TG content (Wang et al., 2013). Conversely, Plin5 knockdown reduces TG accumulation and increases cell lipolysis activity. Wang et al. (2013) observed that global ATGL knockout mice developed severe lipotoxic cardiomyopathy due to massive cardiac TAG accumulation (Drevinge et al., 2016). Acute overexpression of Plin5 in mouse skeletal muscle via adenovirus increased intracellular lipid levels without affecting insulin sensitivity and upregulated genes involved in fatty acid oxidation and mitochondrial function (Drevinge et al., 2016).

**FIGURE 5:**
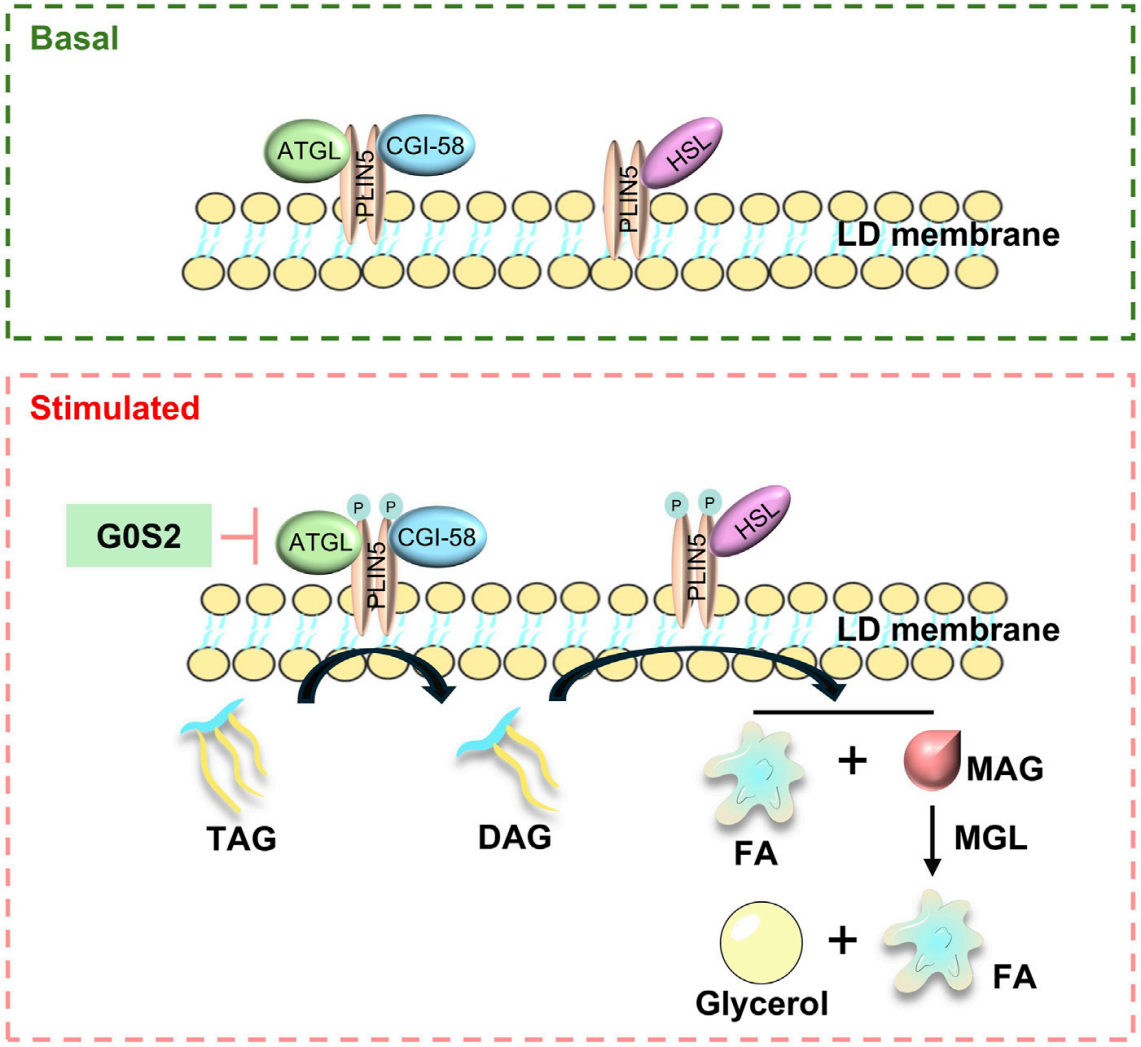
The mechanism of Plin5 regulating lipid droplet decomposition.

Empirical evidence suggests that Plin5 contributes to the stabilization of LDs by inhibiting the uncontrolled hydrolysis of LDs and facilitating the conversion of FFAs into triglycerides for storage, thereby preventing the chronic overloading of mitochondrial capacity. This mechanism ensures the complete metabolism of released FFAs, maintains mitochondrial function, and sacrifices mitochondrial FA oxidation, which indirectly mitigates oxidative stress associated with mitochondrial oxidation. In mice with Plin5 knockout, the use of lipase inhibitors has been shown to ameliorate the depletion of cardiac LDs, further indicating that Plin5 serves as a negative regulatory factor in the hydrolysis of cardiac LDs (Figure 5).

### 3.3 The regulatory mechanism of Perilipin 5

The transcriptional activation and phosphorylation of PAT members are modulated by the elevation of insulin and glucose levels, functioning as transcriptional co-regulators that govern processes including mitochondrial biogenesis and oxidative capacity. Genetic mutations in Plin 5 are intimately linked to the exacerbation of cardiac function following myocardial ischemia, resulting in severe myocardial remodeling and a high mortality rate. The extensive intracellular distribution of Plin 5 points to its involvement in a variety of regulatory mechanisms (Bartholomew et al., 2012). Recent studies have shown that Plin 5 participates in the AMPK/eNOS and PPARα/PGC-1α signaling pathways that are crucial for cardiac metabolic regulation. The PPAR family is subject to regulation by intracellular fatty acids and their derivatives. PPARs have the ability to bind to peroxisome proliferator response elements (PPREs) and retinoid X receptors (RXRs), which can increase the capacity for FA oxidation (Lemberger et al., 1996). PPAR expression is tissue-specific: PPARγ is predominantly found in white adipose tissue (WAT) and macrophages, PPARα is concentrated in the liver and tissues with a high demand for FA metabolism, such as muscle, heart, and kidney, and PPARβ/δ is expressed more broadly. The expression pattern of PPARs is closely aligned with that of PAT proteins. PPARα and PPARβ/δ selectively activate the expression of Plin5, offering a mechanism for tissue-specific activation in mammalian tissues that rely on FA β-oxidation for energy production or thermogenesis.

#### 3.3.1 Plin 5 modulates mitochondrial function through the regulation of mitochondrial-LD connections

Current perspectives suggest that the prerequisite for the oxidation of lipids from LD to mitochondria is the formation of membrane contact sites, termed per droplet mitochondria (PDMs) (Benador et al., 2018). These contact sites are dynamically remodeled in response to various signaling stimuli, playing a role in regulating fatty acid oxidation, the transport of metabolic products, and the generation of neutral lipids (Wang et al., 2011). The N-terminal domain of Plin 5 contains a lipolysis barrier region that promotes FA storage, while its unique C-terminal domain can recruit mitochondria-LDs, migrating to the nucleus to synergistically enhance the function of the co-activator PGC-1α and thereby bolster mitochondrial function (Zhang et al., 2022).

Plin 5 mediates the interaction between LDs and mitochondria and facilitates the transport of fatty acids from LDs to mitochondria through its interaction with the mitochondrial interaction protein, acyl-CoA synthetase Fatp4 (ACSVL4) (Cui and Liu, 2020). Fatp4 is a bifunctional OMM acyl-CoA synthetase that participates in the re-esterification of long-chain fatty acids post-lipolysis, promoting β-oxidation. Knockdown of Fatp4 reduces LD-mito co-localization but does not eliminate it. The absence of Fatp4 disrupts the Plin 5-induced LD-mito contact and FA transport (Miner et al., 2023).

Phosphorylation of Plin 5 at serine 155 and the integrity of the mitochondrial tethering structure are necessary conditions for the transport of FAs from LDs to the mitochondrial matrix (Miner et al., 2023). Plin 5 coordinates the flow of extracellular fatty acids and the contact between LDs and mitochondria to regulate fatty acid oxidation. By mediating the interaction between mitochondria and LDs, Plin 5 balances oxidative energy production, lipid homeostasis, and cellular protection mechanisms in cardiomyocytes (Keenan et al., 2021).

#### 3.3.2 The expression of the Plin5 gene is subject to regulation by the PPAR

PPARα, a pivotal regulator of cardiac substrate switching, is a member of the ligand-activated nuclear receptor superfamily (Barger and Kelly, 2000). The expression of Plin 5 is positively governed by PPARα, a nuclear receptor capable of modulating gene expression upon activation by ligands. Induction of Plin 5 occurs in response to PPARα ligands and enzymes of the mitochondrial FA oxidation pathway, with most targets being under PPARα regulation. Mice lacking PPARα show a marked reduction in Plin5 mRNA levels, whereas PPARα agonists can enhance Plin5 expression. Additionally, the PPARα agonist WY-14643 can rescue Plin5 expression in hypoxic cardiomyocytes (Revuelta-López et al., 2015). PPAR activation increases the uptake and oxidation of fatty acids by modulating the expression of fatty acid transporters and mitochondrial genes involved in FA oxidation. In states of elevated FFA, high-metabolic organs respond by inducing the expression of lipid metabolism-related genes, primarily through the activation of transcription factors such as PPARs. PPARs, upon binding to fatty acid ligands, interact with their heterodimeric partners, the retinoid X receptors, and bind to genomic sequences at PPAR-responsive elements. PPARα and PPARβ/δ are highly prominent in tissues exhibiting elevated FA oxidation rates, whereas PPARγ is most prominently expressed in lipidogenic tissues, such as adipose tissue and the liver. The deficiency of Plin5 exacerbates the progression of cardiac hypertrophy and heart failure induced by pressure overload, concurrently reducing cardiac lipid accumulation and upregulating the levels of PPARα and PGC-1α. These increases are critical for stimulating mitochondrial proliferation and maintaining cellular energy homeostasis.PGC-1α, known as the “master regulator,” oversees the entire metabolic pathway of mitochondrial biogenesis and ATP synthesis. It is downregulated in hypertrophied and failing hearts. Studies using PGC-1α knockout mice have been instrumental in determining the effects of PGC-1α deficiency on cardiac ATP synthesis and contractile reserve (Poulsen et al., 2012).

#### 3.3.3 Plin5 modulates PI3K/Akt phosphorylation, with PKA activation promoting triglyceride lipolysis and fatty acid release

The lipolytic domain of Plin5 orchestrates the storage or mobilization of fatty acids in response to phosphorylation status. By phosphorylating PI3K/Akt, Plin5 mitigates lipolysis of cardiac lipid droplets during ischemia/reperfusion and counters oxidative stress (Zheng et al., 2017). It also serves as a key interaction partner for PGC-1α in response to catecholamine triggering, where PKA activation phosphorylates and activates Plin5, enhancing lipid droplet hydrolysis and FFΑ oxidation efficiency. Plin5 interacts with PGC-1α and SIRT1 in the nucleus to form a transcriptional complex. Phosphorylation deficiency leads to reduced lipolysis and FA oxidation efficiency from triglycerides. Perilipin 5 plays a role in the transcriptional regulation of mitochondrial respiration through nuclear translocation and direct engagement with the PGC-1α mitochondrial gene program. The nuclear function of Plin5 in metabolism may extend beyond cellular autonomy, with future research aimed at determining its potential regulation of SIRT1 targets’ deacetylation status, including p53, FOXO, NFκB, LXR, CRTC2, and SREBP-1 (Zhang et al., 2022).

Zheng et al. (2017) found that ischemia/reperfusion injury increased the expression of phosphorylated PI3K and Akt in mouse heart tissue, while Plin5 knockout decreased these phosphorylated proteins in myocardial tissue, indicating that Plin5 may inhibit PI3K/AKT signalling by suppressing PPARα activation. PKA activation and serine phosphorylation are critical for lipolysis control, with Perilipin 5 enhancing PGC-1α coactivator function by inhibiting SIRT1. The serine at position 155 in Perilipin 5 is identified as a PKA phosphorylation site, and PKA phosphorylation of Perilipin 5 promotes its interaction with ATGL, potentially playing a key role in regulated lipolysis. As a pivotal molecular link, Perilipin 5 integrates the PKA pathway with catecholamine activation and transcriptional regulation of lipid droplet lipolysis, facilitating efficient FA metabolism and mitigating mitochondrial dysfunction (Keenan et al., 2021).

#### 3.3.4 Plin5 plays a pivotal role in regulating the expression levels of Nrf2

Oxidative stress in pancreatic β-cells triggers the activation of Nrf2, as it serves as a principal regulator of the cell’s adaptive response to oxidative stress (Uruno et al., 2015). The transcription factor Nrf2 is capable of safeguarding cells from the detrimental effects of oxidative stress responses. Under normal conditions, Nrf2 is kept inactive through binding to inhibitory proteins like Keap1(Wang et al., 2013). However, during oxidative stress, the oxidation of Keap1 diminishes its binding capacity to Nrf2, leading to the activation of Nrf2 and its subsequent translocation to the nucleus. Here, Nrf2 binds to the ARE and initiates the transcription of downstream genes, which are translated into protective proteins.

Indications suggest that Plin5 may directly influence transcriptional activity by modulating Nrf2 activation and ARE binding. This is supported by the observation that key Nrf2 target proteins, such as GCLC and HO-1, are regulated by Plin5. Moreover, Nrf2 controls the expression of these proteins by binding to ARE sequences in the promoters of antioxidant and detoxifying enzyme genes (Huo et al., 2022).

SIRT1-mediated deacetylation of PGC-1α is known to be a mechanism that controls PGC-1α activity. Plin 5 not only affects the acetylation status of PGC-1α but also activates PGC-1α target genes and inhibits the deacetylase activity of SIRT1.

## 4 The role of Plin5 in REM

In physiological settings, LDs collaborate with Plin5 and a cohort of unknown proteins to establish a connection with mitochondria, thereby exerting control over lipid droplet degradation. However, in the absence of Plin5, the distance between the LDs and the mitochondria increases significantly, about four times more than in normal hearts, leading to a reduction in fatty acid uptake. To maintain myocardial energy metabolism equilibrium, the heart compensates by increasing glucose uptake (Andersson et al., 2017).

Under conditions of heightened cardiac workload and stress, the release of fatty acids from LDs intensifies. Given the heart’s sensitivity to energy metabolism, even slight changes in lipid metabolism can result in a decrease in contractility. Simultaneously, the absence of Plin5 exacerbates the reduction in myocardial substrate metabolic flexibility, causing a precipitous decline in cardiac function.

Cumulative evidence suggests that Plin5 plays a pivotal role in myocardial remodeling by orchestrating the release of fatty acids from triglyceride storage pools into mitochondria and facilitating fatty acid oxidation. The ability of Plin5 to constitutively recruit mitochondria to LDs seems to be a distinctive feature of this protein. Deficiency in Plin5 leads to characteristic defects in mitochondrial segregation, underscoring the protein’s significant relevance to REM.

### 4.1 Perilipin 5 ameliorates cardiac energy metabolic disorders

Perilipin 5 plays a crucial role in the homeostasis of cardiac LDs and is key to understanding the physiological significance of cardiac LDs. Osumi and Kuramoto (2016) created Plin5-deficient mice, which, compared to wild-type mice, lacked LDs in the heart. However, LD synthesis could be induced by transiently perfusing the heart with ATGL inhibitors, highlighting the importance of Perilipin 5 in protecting LDs from lipase attack.

Drevinge et al. (2016) reported that Plin5-deficient hearts exhibit reduced lipid utilization and increased glucose utilization, thereby maintaining energy balance and near-normal cardiac function. There is a triglyceride reduction (TG) accumulation, a decrease in fatty acid uptake, and a preference for binding fatty acids to phospholipids rather than TGs. Plin5-deficient hearts contain a higher proportion of medium-chain TGs and a lower proportion of long-chain and very-long-chain TGs. Zheng et al. (2017) found that Plin5-deficient mice do not show changes in glucose metabolism but have reduced myocardial lipids, suggesting that Plin5 deficiency leads to reduced lipid accumulation and a significant increase in FFAs.

Jian et al. (2023) observed a significant decrease in FA and LD content in myocardial cells of Plin5-deficient neonatal rats, indicating lipolysis in myocardial cells. This study also examined a reduction in glucose unitization in Plin5-deficient myocardial cells, while overexpression of Plin5 in myocardial cells using lentiviral transduction significantly increased glucose uptake. Plin5-null mice were found to oxidize fatty acids more actively than wild-type mice. The absence of Plin5 leads to an increase in the production of ROS in the heart, resulting in a decline in cardiac function over time. This suggests that in the absence of Plin5, there is a lack of control over fatty acid hydrolysis and mitochondrial oxidation, affecting the rate of fatty acid transport and oxidation.

In a myocardial infarction model, researchers observed the accumulation of glycogen in the interventricular septum, indicating that lipid oxidation can still maintain cardiac function. However, in Plin5-null infarcted hearts, there was no glycogen accumulation, and the heart primarily used glucose substrates to maintain energy metabolism, leading to a significant decrease in survival rates (Andersson et al., 2017). This suggests that an increase in glucose utilization cannot maintain cardiac function. In a study of type I diabetes, Plin5-null mice did not show a significant increase in ROS or cardiac dysfunction compared to wild-type mice (Kuramoto et al., 2014).

The absence of Plin5 is associated with an increase in anaerobic glycolysis and the accumulation of lactate in the heart. Lactate, as a key mediator of inflammation and insulin resistance, leads to a reduction in myocardial glucose uptake by mediating AKT phosphorylation (Feng et al., 2022). Concurrently, excessive fatty acid oxidation increases the production of acetyl-CoA and reduces NADH, leading to a reduction in glucose utilization by activating pyruvate dehydrogenase kinase 4 (PDK4). In Plin5-null mice, there is a significant decrease in the expression of GLUT4, a receptor involved in myocardial glucose uptake, along with a substantial reduction in p-AKT phosphorylation (Jian et al., 2023). Overexpression of Plin5 promotes insulin-stimulated GLUT4 translocation to the membrane, enhancing myocardial glucose uptake and utilization.

A Pollak et al. (2015) found that cardiac-specific overexpression of Plin5 in mice leads to extensive lipid accumulation in the heart, similar to the phenotype observed in ATGL-deficient mice. Plin5 overexpression forms a lipolysis barrier at the LD surface, preventing lipase-mediated catabolism. Additionally, compared to ATGL-deficient myocardial cells, Plin5-overexpressing cells exhibit a different morphology, with uniformly sized hypertrophic LDs distributed throughout the cytoplasm and closely associated with mitochondria. These differences suggest that Plin5 overexpression may mildly affect cardiac function, with the study showing that in Plin5-overexpressing mice, each LD is very close to one or more mitochondria, supporting the idea that Plin5 recruits mitochondria and tightly attaches them to the LD surface.

Current research confirms that the absence of Plin5 leads to a decrease in the availability of myocardial metabolic substrates, a decline in cardiac function, and an increase in mortality. Plin5 regulates cardiac energy metabolism by balancing the oxidation of two key metabolic substrates, fatty acids, and glucose, thereby ameliorating cardiac energy metabolic disorders (Andersson et al., 2017).

### 4.2 Perilipin 5 regulates mitochondrial homeostasis and function

Mitochondrial function and structure are critical for energy metabolism and oxidative stress responses. In ischemic cardiomyopathy, the myocardial Z-line and M-line sarcomeres are relatively clear, whereas Plin5-null mice exhibit severe mitochondrial deformation, increased oedema, reduced matrix density, and blurred mitochondrial cristae, indicating significant mitochondrial injury (Zheng et al., 2017). Plin5 deficiency impairs mitochondrial proliferation in ischemic cardiomyopathy, leading to severe mitochondrial injury. This suggests that Plin5 can protect mitochondrial structure and function.

The last 20 amino acids at the C-terminus of Plin5 are observed to mediate physical interactions between LDs and mitochondria in myocardial cells. A reduction in PI3K/Akt phosphorylation levels in the absence of Plin5 exacerbates ischemic cardiomyopathy.

Plin5 deficiency increases the generation of ROS at complexes I and III of the mitochondrial respiratory chain, while also activating PPARα, promoting lipolysis and mitochondrial proliferation, and aggravating myocardial remodeling (Wang et al., 2019). Complex I activity regulates mitochondrial respiration of glutamate. Additionally, it reduces the levels of mitochondrial pyruvate carrier (MPC), decreasing the transport of pyruvate into the mitochondria (Andersson et al., 2017). Jian et al. (2023) found that Plin5-deficient mice have a significant increase in myocardial mitochondria, accompanied by excessive FAO within these mitochondria. The reduction in mitochondrial membrane depolarization observed in Plin5-null mice may be due to changes in the fatty acyl composition of phosphatidylcholine (PC) and phosphatidylethanolamine (PE) in the mitochondrial membrane, with a 40% and 30% change in the fatty acid composition of PC and PE.

Studies indicate that Plin5 activates pathways under nutritional stress, inhibiting apoptosis and excessive autophagy, thereby regulating fatty acid biosynthesis and breakdown to balance lipid metabolism and protect the heart from REM injury. Other research suggests that Plin5 may activate the PI3K/Akt signaling pathway by inhibiting PPARα, with Plin5 cardiac protection achieved through improved mitochondrial energy metabolism, prevention of oxidative stress, enhanced intracellular lipolysis, and regulation of PI3K-related signaling pathways (Zhang et al., 2022).

Phosphorylated Plin5 enhances mitochondrial function by reversing the deacetylase activity of SIRT1, thereby enhancing transcriptional regulation of mitochondrial function and fatty acid metabolism. It regulates mitochondrial proliferation and oxidative activity, with the last 20 C-terminal amino acids playing a role in mitochondrial anchoring and enhancing respiratory capacity (Kien et al., 2022). Concurrently, it upregulates mitochondrial function-related genes to compensate for the mitochondrial deficits caused by myocardial remodeling (Tan et al., 2019).

### 4.3 Perilipin 5 inhibits oxidative stress and myocardial cell apoptosis

Oxidative stress is a precursor to myocardial remodeling, arising from an imbalance between the production of ROS and antioxidant capacity within cells (Cinato et al., 2024). Myocardial diseases trigger oxidative stress due to dysregulation of energy metabolism, leading to calcium handling defects, arrhythmias, and subsequently increased apoptosis, which further exacerbates myocardial remodeling. Strategies that regulate mitochondrial oxidative stress and mtRNA translation are considered effective treatments for preventing myocardial remodeling and heart failure (Tsutsui et al., 2009).

Plin5 affects myocardial remodeling by regulating the activity of signaling pathways related to cell proliferation and apoptosis. Changes in Plin5 expression and function impact the balance between myocardial cell proliferation and apoptosis. Plin5 activates the PI3K/Akt signaling pathway24, which can be regulated by ROS, thereby controlling apoptosis and inflammatory responses. The conversion of FAO to ATP is accompanied by ROS production (Wang et al., 2015).

Studies have shown that in high-fat-induced mouse models, myocardial mitochondrial ROS levels increase while oxygen consumption remains unchanged, and myocardial LD content decreases. ROS, acting as intracellular second messengers, can further promote myocardial remodeling through DNA injury, protein oxidation, cellular dysfunction, and increased myocardial cell apoptosis (Rababa’h et al., 2018).

Plin5 modulates the Akt/GSK^-^3β/Nrf2 signaling pathway to prevent apoptosis, ROS production, and inflammatory responses induced by high glucose (HG) in diabetic nephropathy (Zheng et al., 2017). By assessing lipid peroxide levels, it was found that Plin5^−/−^ mice hearts significantly promoted the generation of ROS during I/R injury. Isolated myocardial cells from Plin5^−/−^ mice demonstrated more active oxidation of FFA and ROS recruitment compared to wild-type (WT) mice (Revuelta-López et al., 2015). Studies on hypoxic myocardial cells also showed that the absence of Plin5 significantly increased FFA and ROS levels (Revuelta-López et al., 2015). ROS-induced oxidative injury leads to myocardial cell dysfunction, and echocardiographic assessments revealed that Plin5^−/−^ mice exhibited a more pronounced decline in cardiac function at an advanced age compared to WT mice. Continuous treatment with N-acetylcysteine (NAC) mitigated the decline in cardiac function and reduced lipid peroxide levels, indicating that Plin5 crucial role in the heart is to inhibit excessive ROS production by protecting LDs, isolating FAs, and reducing mitochondrial FA oxidation (Wang et al., 2019). Overexpression of Plin5 leads to oxidative stress and triggers the upregulation of the Nrf2 antioxidant pathway, which plays a protective role in the heart. The activation of the Nrf2 antioxidant pathway can prevent the progression of Plin5-overexpressing hearts to heart failure.

It is speculated that the increased ROS production in the hearts of Plin5 knockout mice is due to excessive FA entering mitochondrial oxidative metabolism. In contrast, the ROS production observed in Plin5-overexpressing hearts may result from the excessive formation of LDs-induced endoplasmic reticulum stress or structural changes in mitochondria due to the proximity of LDs (Kuramoto et al., 2012). Overall, mutations such as Plin5 knockout or overexpression contribute to the development of dysfunction and cardiac steatosis (Wang et al., 2013).

Plin5^−/−^ mice amplify I/R-mediated ROS injury, with the myocardial ischemic and hypoxic state triggering changes in energy metabolism substrates and an intensified demand for oxygen, which further promotes ROS accumulation (Zheng et al., 2017). MDA, a cytotoxic product of ROS-induced lipid peroxidation, serves as an indicator of cellular injury levels. A marked elevation in MDA levels in Plin5^−/−^ mice suggests that Plin5 plays a protective role by reducing ROS production in myocardial cells (Zheng et al., 2017).

Excessive cytoplasmic FAs elicit β-oxidation, which in turn generates ROS. ROS at moderate to high concentrations can induce apoptosis and, in certain cases, oxidative stress can precipitate necrosis. MDA, a cytotoxic byproduct of ROS-induced lipid peroxidation, is a reliable indicator of the degree of lipid peroxidation. SOD, a vital antioxidant enzyme, can neutralize ROS in the body, diminish ROS-induced cellular injury, and aid in the restoration of affected cells (Wang et al., 2013).

Perilipin 5 in the myocardium fosters the formation of LDs and mitigates FA oxidation, thereby shielding the myocardium from lipotoxic injury. Recent research has shown that Perilipin 5 plays a pivotal role in preserving cardiac function by preventing excessive lipolysis, FA oxidation, and oxidative stress.

Therefore, a deficiency in Plin5 intensifies FA oxidation, leading to an augmented production of ROS, which in turn aggravates ROS-mediated injury and increases oxidative stress in the heart. The accumulative effects of heightened oxidative stress can result in a further weakening of cardiac function.

### 4.4 Perilipin 5 plays a crucial role in regulating myocardial hypertrophy and fibrosis

Pathological hypertrophy of myocardial cells is associated with marked changes in energy metabolism and a significantly heightened risk of heart failure. Pressure overload-induced hypertrophy is an adaptive response aimed at reducing cardiac wall stress and oxygen consumption. PPAR-γ agonists have been demonstrated to inhibit protein synthesis and prevent hypertrophy induced by pressure overload.

Initial research showed that myocardial-specific overexpression of Perilipin 5 promotes cardiac hypertrophy without impacting cardiac function. Physiological hypertrophy is considered harmless and reversible, with Plin5 improving the maintenance of cardiac physiological function in a remodeling environment (Cinato et al., 2023). Wang et al. (2013) observed that cardiac-specific overexpression of Plin5 in mice led to cardiac steatosis, mild dysfunction, reduced mitochondrial function, and concentric left ventricular hypertrophy. Mice lacking Plin5 exhibit reduced cardiac metabolic flexibility and impaired function following cardiac stress, such as myocardial infarction. Reduced cardiac Plin5 levels are correlated with decreased human cardiac function.

Myocardial fibrosis, a definitive feature of pathological cardiac remodeling, is manifested by the excessive accumulation of extracellular matrix (ECM) within the myocardial interstitium. Activated fibroblasts are the predominant source of ECM during myocardial fibrosis. The transformation of fibroblasts into myofibroblasts, which secrete ECM, is a pivotal cellular and biological alteration in the fibrotic process. The proliferation and activation of fibroblasts in fibrotic myocardial tissue may result from direct actions of fibroblast mediators or from upstream stimulation by other cells, which secrete cytokines, growth factors, and other pertinent proteins. TGF-β is the most critical cytokine in myocardial fibrosis, with the ability to stimulate the transcription of fibrosis-related genes and the expression of other profibrotic cytokines via the activation of the Smad2/3 signaling pathway.

Lactic acid, a signaling molecule mediating various pathological and physiological processes, has been shown in recent studies to potentially regulate myocardial hypertrophy by restoring the lactylation of α-MHC and its interaction with titin. A study involving Plin5 knockout mice found a significant increase in lactic acid accumulation, which, by inhibiting the pyruvate carrier (MPC), directed glucose towards lactate metabolism to drive myocardial cell hypertrophic growth, leading to severe myocardial hypertrophy. This suggests a role for Plin5 in the regulation of myocardial hypertrophy.

Lactic acid is a signaling molecule that mediates many pathological and physiological processes (Dai et al., 2020). Recent studies suggest that administering lactic acid or inhibiting its efflux may regulate myocardial hypertrophy by restoring the lactylation of α-MHC and the interaction between α-MHC and titin. A study observing Plin5 knockout mice found a significant increase in lactic acid accumulation, and it was proposed that Plin5 deficiency drives myocardial cell hypertrophic growth by inhibiting the MPC and directing glucose towards lactate metabolism, leading to severe myocardial hypertrophy. This indicates the involvement of Plin5 in the regulation of myocardial hypertrophy (Cluntun et al., 2020).

## 5 Conclusion and perspectives

Plin5, as a key protein in the process of myocardial remodeling, represents a promising therapeutic target for the treatment of myocardial diseases associated with cardiac metabolic disorders. Serving as a bridge between mitochondria and FFA metabolism, the targeted regulation of energy metabolism changes is of utmost importance. Plin5 ensures favourable fuel supply and directs FFA to LD storage, addressing the energy metabolic disturbances induced by myocardial remodeling.

In the realm of REM, Plin5 modulates the phosphorylation levels of PI3K/Akt and the mitochondrial-LD connection, collectively influencing FFA utilization. It also mediates the expression of Nrf2 to regulate mitochondrial stability and function, inhibiting oxidative stress and myocardial cell apoptosis and preventing myocardial hypertrophy and fibrosis caused by metabolic disturbances. The PAT family exhibits mutual regulation and compensation capabilities; for instance, in Plin2 knockout mice, there is an increase in Plin3 and Plin5 levels, both of which are involved in regulating LD turnover and the acquisition of lipases. Strategies targeting mitochondrial oxidative stress and mtRNA translation are considered effective therapeutic approaches.

Mir-370 and Mir-292-5p can protect myocardial tissue from I/R injury by activating Plin5. The use of Plin5-coated artificial LDs facilitates cellular uptake, significantly inhibiting ROS accumulation and the cytotoxicity caused by excessive FFA. Overexpression of Plin5 in the heart leads to steatosis, mild mitochondrial oxidative defects, and an increase in ROS, yet it prevents the occurrence of cardiac dysfunction. This suggests that Plin5 counteracts adverse factors during myocardial remodeling to maintain heart function, which has a preventive effect on heart failure induced by myocardial remodeling. Overall, these findings hint at the role of Plin5 in regulating the progression of REM.

Given the complex regulatory mechanisms of the PAT protein family, targeting the feedback regulation pathways of Plins is key. Simultaneously, the interplay between cardiac energy metabolism pathways, substrate supply, and utilization is challenging to study using traditional methods. Bioinformatics approaches (genomics, transcriptomics, proteomics, metabolomics, and systems biology) are powerful tools for regulating Plin5 and addressing cardiac energy metabolism, precisely resolving metabolic disturbances in REM hearts.
